# The Ilocos Norte Communities against Rabies Exposure Elimination Project in the Philippines: Epidemiological and Economic Aspects

**DOI:** 10.3389/fvets.2017.00054

**Published:** 2017-04-24

**Authors:** Loida M. Valenzuela, Sarah I. Jayme, Anna Charinna B. Amparo, Louise H. Taylor, Maria Pinky Z. Dela Cruz, Dianne A. Licuan, Rosebelle Gamal-Bitao, Louis H. Nel

**Affiliations:** ^1^Provincial Veterinary Office, Ilocos Norte, Philippines; ^2^Global Alliance for Rabies Control, Santa Rosa, Philippines; ^3^Global Alliance for Rabies Control, Manhattan, KS, USA; ^4^Department of Microbiology and Plant Pathology, University of Pretoria, Hatfield, South Africa

**Keywords:** canine rabies, rabies elimination, dog vaccination, Philippines, anti-rabies vaccine costs

## Abstract

As canine rabies control in Africa and Asia transitions from research-led proof-of-concept studies to government-led programs for elimination, experience and evidence of their impact and costs must be shared for the benefit of future programs. The Ilocos Norte Communities against Rabies Exposure project was implemented in April 2012 by the provincial veterinary and health offices and supported by many other partners. It delivered a comprehensive dog vaccination program and increased awareness of the need for postexposure prophylaxis (PEP), aiming to eliminate human and animal rabies cases from Ilocos Norte by 2015. Prior to the intervention, confirmed rabies cases in dogs were between 19 and 50 per year (2008–2011). The primary outcome of the project was a reduction in rabies cases in both dogs and humans to 0 in 2014 and 2015, which has subsequently been maintained. Animal bite consultations increased significantly during the project. Economic data for the dog vaccination and PEP components of the project were collated for two sites: Laoag City (an urban setting) and Dingras Municipality (a rural setting) between 2012 and 2014. The average programmatic cost of vaccinating each dog was $4.54 in Laoag City and $8.65 in Dingras, and costs fell as the project reached more dogs. The average costs of providing PEP were $69.72 per patient and $49.02 per patient for the two sites, respectively, again falling as the project reached more people. External donor contributions contributed less than 20% of dog vaccination costs and less than 1% of PEP costs. The project demonstrated that rabies elimination can be achieved in a short period of time, with concerted effort across multiple sectors. A lack of clear dog population estimates hampered interpretation of some aspects of the programme. From 2016, the provincial government has assumed complete responsibility for the programme and must now continue the vaccination and surveillance efforts. Although safeguards are in place, reintroduction from surrounding areas remains a threat, and vigilance must be maintained.

## Introduction

Most canine rabies-endemic countries have been implementing rabies control efforts for decades. However, incomplete provision of postexposure prophylaxis (PEP) to dog bite victims, a lack of comprehensive dog vaccination campaigns, and weak collaboration between animal and human health sectors have prevented elimination of the disease (1). An estimated 59,000 human deaths a year still occur as a result of canine rabies (2).

Over the last decade, research-led proof-of-concept studies have provided theoretical and practical evidence that canine rabies elimination is feasible even in Africa and Asia where the disease still exerts a heavy burden (3–6). Success at scale has been achieved in Latin America, where major canine vaccination efforts have led to the elimination of the public health threat in many countries (7). In Africa and Asia, however, only a small number of countries or provinces have enacted effective mass dog vaccination programmes, for example, in KwaZulu-Natal in South Africa and SE Tanzania (5), Sri Lanka (8), and Bohol (9) and the Visayas region in the Philippines (5).

Research-led disease interventions focus on detailed data collection to gain an understanding of disease control mechanisms. In contrast, government-led interventions tend to implement accepted techniques at a larger scale, seeking to deliver health benefits but with less detailed assessment of the actual impact. Particularly because of the zoonotic nature of the disease, there is a need to document the ways in which rabies programs have been structured, the partners that have contributed, and the lessons learned.

## Background and Rationale

Despite the 2008 Call for Action toward the Elimination of Rabies in the ASEAN Member States by 2020 (10), little progress toward this goal has been achieved. In December 2015, an ambitious global goal of an end to dog-mediated human rabies by 2030 was set (6). The global framework for rabies elimination developed at the December 2015 meeting is based on five essential program pillars: socioeconomic, technical, organization, political, and resource, to reflect the range of interventions necessary to ensure successful elimination (11). Lessons learned from government-led programs in how they implement these pillars and their successes and costs will benefit rabies elimination programs planned elsewhere and enable the faster scale up of programs necessary to reach rabies elimination.

A previous rabies prevention and elimination project, which was implemented from 2007 to 2009 on the island of Bohol in the Philippines, proved effective within 2 years (9). The same multisectoral model was adapted for Ilocos Norte to test whether rabies could be eliminated in a province bordered by areas still endemic for canine rabies. All activities implemented were anchored on the Philippines National Rabies Prevention and Control Program, which is well supported by legislation.

## Essential Elements of the Intervention

The Ilocos Norte Communities against Rabies Exposure (CARE) project was implemented from April 2012 until September 2016. The multisectoral program was spearheaded by the Provincial Rabies Control Committee, with the Provincial Veterinary Office (PVO) collaborating with the Provincial Health Office (PHO) and other local agencies such as interior and local government, the Department of Education, local police, medical associations, and universities. A donation of animal rabies vaccines was provided by World Animal Health Organisation (OIE) through the OIE Regional Vaccine Bank for Asia and in kind support was received from the Department of Agriculture-Bureau of Animal Industry as well as provincial and local government units. Technical input was provided by the Global Alliance for Rabies Control through the support of the UBS Optimus Foundation.

The project’s goal was to improve the existing rabies control approach and eliminate canine-mediated human rabies cases in the province by 2015. The interventions not only focused on comprehensive mass dog vaccination aiming to reach 70% coverage across the province and raising community awareness of the risk of rabies in a variety of school- and community-based settings but also involved additional training in surveillance and diagnosis. Barangay health workers and other volunteers were trained to support the rabies project and paved the way to increasing the number of dogs vaccinated during the project intervention phase. Educational interventions included an early childhood intervention program to teach animal bite prevention in preschool day care centers, integration of rabies information into the grade school curriculum, training of rabies speakers for the community, and various media outreach (separate manuscript in preparation). The activities of the full program are summarized in Table 1 and reflect in 2015 the inclusion of a more rigorous monitoring system and a more extensive training program to ensure sustainability of the program after its completion. To intensify the one health surveillance system, a multisectoral reporting mechanism was institutionalized such that highly suspicious rabid dogs located following bite reports were placed under observation. After the end of the CARE project period, the local government partners are continuing to implement the program’s initiatives to ensure that the public health benefits are maintained.

**Table 1:** **Rabies prevention and control activities implemented as part of the Ilocos Norte Rabies project**.

Activities	Implemented by	2012	2013	2014	2015
Establishment of Provincial Rabies Control Committee	PVO, PHO				
Intersectoral collaboration (universities, animal welfare organization, professional groups, and media)	PRCC				
Mobilize BRBVs	PVO				
Train BRBVs as vaccinators	PVO, GARC				
Mass dog vaccination	PVO, CVO, MAO, DA-BAI, OIE Vaccine Bank, GARC, UPCVM				
Dog registration	PVO, CVO, MAO				
Dog Population Survey	PVO, GARC, MAO, PDRRMO, HSI				
Human Rabies Case investigations	PHO				
Institutionalization of “One Health” response system	PVO, PHO, GARC				
Laboratory diagnosis training and biosafety	PVO, RITM, GARC				
Training on use of nets and humane handling of dogs	PVO, HSI, Cebu City DVMF, GARC				
Dog rabies case investigation training	DARFU I, PVO				
Rabies Speakers’ Bureau (to increase community participation and advocate for rabies control)	PVO, MMSU, MAO, MHO, GARC				
Integration of rabies in the grade school curriculum	PVO, PHO DepED, GARC				
Early Childhood Intervention Program in Pilot Day Care/Pre-School Centers	PVO, PSWD GARC, MMSU				
Provincial quiz bee on rabies	PVO, PHO, DepEd				
Information campaign to strengthen border control	PVO, PHO, DA-BAI, DOH, GARC				
Region I Rabies Summit (to disseminate achievements)	PVO, PHO, DA-BAI, DARFU I, CHD Region I, GARC				
Policy advocacy for local government executives	PRCC				
Media Awareness Workshop	GARC				
Awards for best local government rabies implementers	PRCC				
Community-based survey (including Knowledge, Attitudes and Practice)	GARC, PVO, MMSU				

*Shaded sections indicate the years that the activities were completed*.

*Key to project partners: Cebu City DVMF, Cebu City Department of Veterinary Medicine and Fisheries; CHD Region I, Centre of Health for Development Region I; CVO, City Veterinary Office; DA-BAI, Department of Agriculture Bureau of Animal Industry; DARFU I, Department of Agriculture Regional Field Unit; DepED, Department of Education; GARC, Global Alliance for Rabies Control; HSI, Humane Society International; MAO, Municipal Agriculture Office; MHO, Municipal Health Office; MMSU, Mariano Marcos State University; OIE, World Organisation for Animal Health; PDRRMO, Provincial Disaster Risk Reduction Management Office; PHO, Provincial Health Office; PRCC, Provincial Rabies Control Committee; PSWD, Provincial Social Welfare Development; PVO, Provincial Veterinary Office; RITM, Research Institute for Tropical Medicine; UPCVM, University of the Philippines College of Veterinary Medicine; BRBV, Barangay Bantay Rabies sa Barangay Volunteers*.

Here, we present the key epidemiological findings and an economic evaluation to determine the programmatic costs of the dog vaccination and human PEP provision.

## Methods

### Study Site

Ilocos Norte is a province of the Philippines in the Ilocos Region, located at the northwest corner of Luzon Island. Its total land area is 3,622.91 km^2^ (12). It is bounded by four provinces to the east and south and the South China Sea to the west. There are 13 mountains in the province, almost all in the southeastern portion. It has 557 barangays (the smallest administrative division) in 21 municipalities and 2 cities.

The human population of Ilocos Norte in the 2015 census was 593,081, a density of 170 people/km^2^ (13). The majority (81.3%) of the population resides in rural areas. The province has one of the lowest poverty incidences in the Philippines, at 12.3% in 2015 (14), and the average annual family income in 2012 was Philippine pesos (PHP) 254,923 ($5,275 US at current exchange rates) (15).

Legislative support for rabies control is very good in the Philippines, with the Republic Act 9482 (the Anti Rabies Act of 2007) providing a comprehensive legal framework for the implementation of rabies prevention measures from national to local levels. Provisions of the law include the target of rabies elimination in the country by 2020; policies on mass dog vaccination, responsible pet ownership, and access to PEP; roles of different national agencies and local government units; and penalties for violators. The Provincial Government of Ilocos Norte enacted Provincial Ordinance no. 82–97 (Ordinance Governing Rabies Control in Ilocos Norte) in 1997, which was revised in 2001 and 2008 to adapt to the Anti-Rabies Act of 2007. All municipalities, cities, as well as two barangays also have respective ordinances on rabies.

Before the project implementation, rabies control efforts involved sporadic vaccination campaigns in response to rabies outbreaks, most recently in 2010, together with provision of subsidized PEP at animal bite treatment centers. Surveillance data showed 19–50 confirmed rabies cases in dogs and around 2 human deaths per year in the province from 2008 to 2011.

### Surveillance Data

Surveillance data from the project site were extracted from the relevant national databases in the Philippines. Data on numbers of animals tested and confirmed canine rabies cases were obtained from the Regional Animal Disease Diagnostic Laboratory 1. Dog brain testing was carried out using the gold standard fluorescent antibody test at the Provincial Animal Rabies Diagnostic Laboratory of the PVO. Starting in 2016, Municipal Agricultural Offices are required to submit monthly negative monitoring reports to the PVO. Field staff at the municipal level list the barangays they have visited in a month and indicate in the report that they have not encountered a highly suspect rabid animal in the places they have visited.

Data on human rabies cases was obtained from the Epidemiology Bureau of the Department of Health. Cases are classified as suspect, probable, or confirmed. Suspect cases are those presenting with clinical signs of furious and paralytic rabies leading to coma and death. Probable cases are suspect cases with contact with suspected rabid animals. Confirmed cases are those with laboratory confirmation, which is not yet fully implemented in the Philippines. Data on the number of Animal Bite Treatment Centre (ABTC) consultations were obtained from the Center for Health Development for Region I. Bite consultations are collated from eight ABTCs across Ilocos Norte by the PHO quarterly and then submitted to the Region 1 Center for Health Development.

### Dog Vaccinations, Dog Population, and Vaccination Coverage

As part of the CARE project, mass dog vaccinations were carried out in a rolling program from March to November each year from 2012, using a fixed point and door-to-door vaccination strategy. The province takes advantage of the publicity and awareness campaigns of the March national Rabies Awareness Month (preceding the bite incidence peak in summer) to start the mass dog vaccination campaigns, which end before the December holiday period in the Philippines. The strategy implemented in a particular location was chosen to best suit the geographic setting and the preferences of the community. Records of the number of dogs vaccinated in each barangay each year were collated by the Ilocos Norte PVO.

Several methods of dog population estimation have been used in Ilocos Norte. Before 2013, the dog population was estimated based on vaccination activities in high-risk areas (those with human rabies cases and city centers) and assuming a 1:10 dog:human ratio in all other areas. This strategy was improved by obtaining the number of dogs from city and municipality dog registration records. In 2014, a community-based survey (CBS) conducted house-to-house interviews utilizing cluster sampling of households and completing a structured questionnaire. This determined the proportion of households that owned dogs, determined the mean number of dogs owned, and provided an estimate for the dog:human ratio.

A more comprehensive dog population survey (including both owned and unowned dogs) was conducted early in 2016. This employed household surveys and dog counts conducted in 36 1 km × 1 km randomly chosen grids, corrected for incomplete detectability and incomplete coverage of roads within grids, where relevant. Corrected dog counts were then compared to human density data from Oak Ridge National Laboratory’s Landscan data layer for the Philippines (2013) to give dog:human ratios for three different density categories (0–1,000 people/km^2^, 1,000–5,000 people/km^2^, and >5,000 people/km^2^). From here, the total dog population of the province was estimated.

Overall vaccination coverage was estimated indirectly by dividing the total number of dogs vaccinated by the total population as assessed by the different dog population surveys.

### Economic Data

Economic data for the provision of the dog vaccination program and PEP administration were collated for two sites: Laoag City (highly urban) and Dingras Municipality (rural), for the years 2012–2014. Costs were obtained by interviewing key personnel at the PVO, City Veterinary Office, and Municipal Agriculture Office of Dingras for the dog vaccination costs and the PHO, City Health Office, Gov. Roque B. Ablan Sr. Memorial Hospital ABTC, and Dingras District Hospital ABTC for the costs of providing PEP to bite victims.

The costs of (salaried) personnel, awareness and social mobilization (including volunteer training), vaccines, rabies immunoglobulin, consumables, and other cost categories were used to calculate the programmatic cost of vaccinating each dog and providing each course of PEP, as well as to indicate the division of costs between different stakeholders.

One of the three ABTCs in Laoag City was located within a government-operated hospital, but managed by a private health provider in 2012 and 2013. The private provider covered some personnel costs during this time, but the remaining costs were paid by the government hospital. In 2014, the government took over the ABTC’s full management, which has remained the case since. The remaining facilities were fully managed by the government throughout the time period analyzed.

Costs were converted from PHP to US dollars (USD) using an exchange rate of 1 PHP = USD0.022.

## Results

### Surveillance of Animal Cases, Human Bites, and Human Cases

During the course of the project, between 32 and 48 dog brains were sampled each year (Table 2). In 2012, eight dogs (23%) tested positive, which fell to 1 (2%) in 2013 and then 0 subsequently. These figures represent significant reductions from the average of 35.5 confirmed dog cases and the average of 38.8% samples proving positive for rabies from 2008 to 2011 (Table 2). The program of monthly negative monitoring reports started in 2016 has yielded no further case reports.

**Table 2:** **Indicators of animal and human rabies risks over the project period**.

	Prior to project implementation				After project implementation			
	2008	2009	2010	2011	2012	2013	2014	2015
**Animal and human cases**								
Dog brain samples tested	123	87	90	66	36	45	48	32
Confirmed dog cases	50	29	44	19	8	1	0	0
% of samples positive	40.7	33.3	48.9	28.8	22.2	2.2	0	0
Suspect human cases	2	1	2	2	2	1	0	0
Annual incidence of suspect human cases per 100,000 (*N* = 568,017)	0.352	0.176	0.352	0.352	0.352	0.176	0.000	0.000
Patients seeking animal bite consultations	897	954	1,475	2,015	3,070	3,571	5,908	5,520
Number of children seeking animal bite consultations (%)	Not available				1,265 (42)	1,378 (43)	2,052 (40)	1,394 (41)
**Dog vaccinations**								
Number of barangays reached by the vaccination campaign	329	342	381	266	340	420	485	447
% Barangays reached (*n* = 557)	59.1	61.4	68.4	47.8	61.0	75.4	87.1	80.3
Number of dogs vaccinated	12,044	12,203	28,581	12,066	23,539	39,647	38,722	36,460
% Dogs vaccinated (assuming *n* = 76,628)^a^	15.7	15.9	37.3	15.7	30.7	51.7	50.5	47.6
% Dogs vaccinated (assuming *n* = 149,748)^b^	8.0	8.1	19.1	8.1	15.7	26.5	25.9	24.3
% Dogs vaccinated (assuming *n* = 278,691)^c^	4.3	4.4	10.3	4.3	8.4	14.2	13.9	13.1

*A child is defined as anyone 15 years or younger*.

*^a^Concluded from dog censuses in 2013*.

*^b^Concluded from community survey in 2014*.

*^c^Concluded from comprehensive dog population assessment in 2016*.

According to the national clinical criteria, one or two suspect human cases per year were diagnosed from 2008 to 2011. Following the implementation of the project, two suspect human cases were reported in 2012 and one in 2013. Since then, no cases have been reported, as of October 2016 (Table 2). This corresponds to an annual incidence (per 100,000) of 0.352 human cases in 2012, falling to 0.176 in 2013 and then to 0 from 2014 onward (Table 2).

Animal bite consultations rose from 3,070 in 2012 to a peak of 5,908 in 2014 and then fell to 5,520 in 2015, and 40–43% of patients were younger than 15 years (Table 2). The proportion of bites attributed to dogs was 83–89%, and other bites were from cats and various small mammals. Likely due to increased rabies awareness efforts by the Philippines government and an increase in the number of ABTCs in the province, the number of ABTC consultations had been rising prior to the CARE project’s implementation. However, during the project, the rate of increase in uptake of these services rose markedly (Table 2).

### Dog Vaccinations and Coverage

Before the project, around 12,000 dog vaccinations were conducted per year, apart from in 2010 when an emergency intervention in response to rising rabies cases increased this to over 28,000 (Table 2). As part of the CARE project, mass dog vaccination campaigns increased the number of barangays covered from 61.0% in 2012 to 75–87% in subsequent years and the number of dogs vaccinated to an average of 38,276 each year (Table 2).

The vaccination coverage achieved was not routinely collected as part of the vaccination campaign, leaving only indirect assessments based on the estimated dog population. The total dog population was initially estimated in 2012 at 35,000, based on numbers from previous vaccination campaigns in high-risk areas, and a dog:human ratio of 1:10 elsewhere. Subsequently, dog censuses were conducted by the barangay rabies volunteers every first quarter of the year before the start of the annual mass dog vaccination. These records yielded estimates of the owned dog population of 76,628 for 2013, 68,655 for 2014, and 63,815 for 2015.

Realizing that the dog population may have been underestimated, CBS was conducted in 2014. These surveys found that 65.9% of houses owned dogs, with 71% of those houses owning one or two dogs and 5% more than four dogs. The calculated dog:human ratio was 1:3.8, generating an estimated owned dog population of the province of 149,748, of which 67% were free roaming.

Finally, a rigorous dog population survey in 2016 calculated the unowned, owned roaming, and owned confined dog populations for each of three human density categories. For the human density categories <1,000 people/km^2^, 1,000–5,000 people/km^2^, and >5,000 people/km^2^, the estimated total dog:human ratios calculated were 1:2.03, 1:2.55, and 1:2.25, respectively (see Table S1A in Supplementary Material). It concluded an overall dog:human ratio of 1:2.24, a total owned dog population of 217,469 (53% free roaming), and an additional 61,222 unowned free-roaming dogs, giving a total dog population estimate of 278,691 (overall 36% confined by owners) (Table S1B in Supplementary Material).

The very different dog population estimates mean that the vaccination coverage achieved by the project is very hard to calculate with certainty. Using the estimated dog population from the 2013 dog census (76,628), the average coverage level from 2013 to 2015 was 50.0%, but using the estimated population from the 2014 CBS, the average coverage was 25.6%, and using the 2016 comprehensive dog population survey data, the average coverage was just 13.7% (Table 2).

Alternative estimates of vaccination coverage came from household surveys conducted during the 2016 rigorous dog population survey, which suggested that 38.8% of owned dogs (and therefore likely around 30% of all dogs) had been vaccinated against rabies during the previous year. This is consistent with the 38% vaccination coverage of owned dogs estimated from the 2014 CBS.

### Cost per Dog Vaccinated

The programmatic cost of vaccinating dogs (including vaccine, salaries, equipment, and other costs) and the derived cost per dog are presented in Table 3. For Laoag City, the average vaccination cost per dog fell from $7.03 to $3.09 over the 3 years of the project as the number of dogs vaccinated increased (overall cost = $4.54 per dog; Table 3). Costs for the Dingras Municipality (overall cost = $8.65 per dog) also fell when higher numbers of dogs were vaccinated after 2012, but were on average higher than in Laoag City, likely due to the lower number of dogs vaccinated, especially in 2012 (Table 3).

**Table 3:** **Programmatic costs for dog vaccination and postexposure prophylaxis (PEP) provision in Laoag City and Dingras Municipality**.

	2012	2013	2014	Overall
**Dog vaccination costs for Laoag City**				
Total cost of dog vaccination (PHP)	1,110,365	1,040,485	1,054,202	3,205,053
Total cost of dog vaccination (USD)	24,428	22,891	23,192	70,511
Number of dogs vaccinated	3,476	4,527	7,513	15,516
Cost/dog (USD)	7.03	5.06	3.09	4.54
**Dog vaccination costs for Dingras Municipality**				
Total cost of dog vaccination (PHP)	591,155	751,045	648,821	1,991,021
Total cost of dog vaccination (USD)	13,005	16,523	14,274	43,802
Number of dogs vaccinated	726	2,982	1,357	5,065
Cost/dog (USD)	17.91	5.54	10.52	8.65
**PEP costs for Laoag City**				
Total cost for PEP (PHP)	2,156,857	2,297,622	2,755,215	7,209,695
Total cost for PEP (USD)	47,451	50,548	60,615	158,613
Number of PEP doses provided	2,295	2,402	3,397	8093.13
Number of patients vaccinated	636	726	913	2,275
Average doses/patient	3.61	3.31	3.72	3.56
Cost per PEP dose (USD)	20.68	21.05	17.85	19.60
Cost per patient (USD)	74.61	69.62	66.39	69.72
**PEP provision cost for Dingras Municipality**				
Total cost for PEP (PHP)	183,976	404,390	755,128	1,343,494
Total cost for PEP (USD)	4,047	8,897	16,613	29,557
Number of PEP doses provided	202	507	1,207	1,917
Number of patients vaccinated	63	158	382	603
Average doses/patient	3.21	3.21	3.16	3.18
Cost per PEP dose (USD)	20.01	17.54	13.76	17.11
Cost per patient (USD)	64.25	56.31	43.49	49.02

*PHP, Philippine pesos; USD, US dollars*.

*The overall column shows the totals from 2012 to 2014, except for the costs per dog, per patient or per dose where the mean is given*.

**Figure 1:**
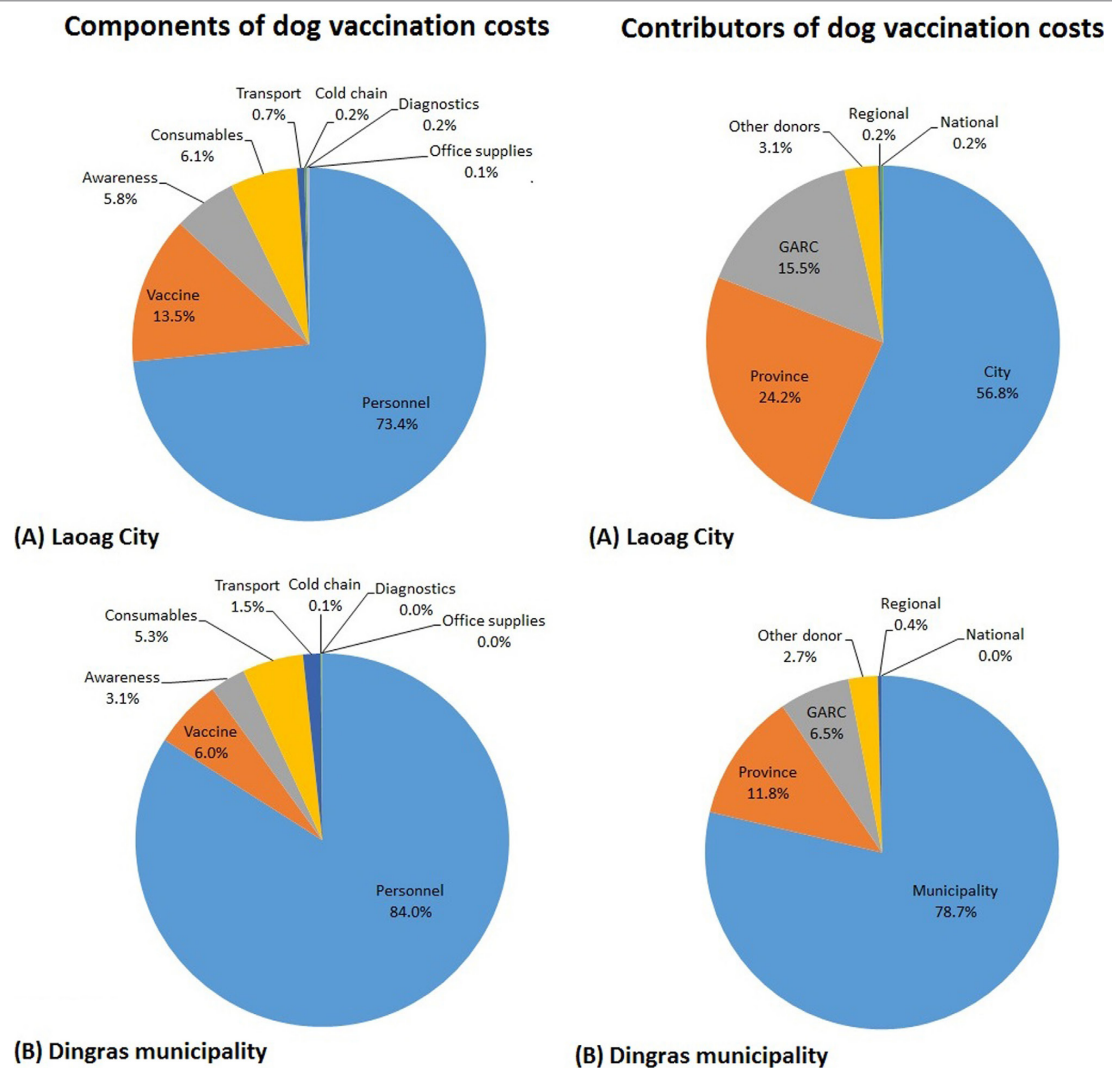
**The cost of vaccinating dogs divided into the different components and between the different project contributors for (A) Laoag City and (B) Dingras Municipality from 2012 to 2014**.

The breakdown of cost into components and by project partner is provided in Table S2 in Supplementary Material and summarized in Figure 1. Personnel costs formed the bulk of the expenditure (73.4% for Laoag City and 84.0% for Dingras Municipality), followed by the cost of the vaccine (13.5% for Laoag City and 6.0% for Dingras Municipality) with consumables and awareness activities the next highest costs (Figure 1).

The majority of funding for dog vaccination in Laoag City came from the city and provincial governments (56.8 and 24.2%, respectively; Figure 1). For Dingras Municipality, the bulk of the funding was provided by the municipality and the provincial government (78.7 and 11.8%, respectively). In each case, these were the main sources of funding for the personnel costs (Table S2 in Supplementary Material). National and provincial governments provided a very small proportion of the total funds spent, and donors (Global Alliance for Rabies Control and the OIE vaccine donation) provided a total of 19 and 9% of the dog vaccination costs for Laoag City and Dingras Municipality, respectively.

### Costs per Patient Provided with PEP

The programmatic cost (inclusive of salaries and other components necessary to deliver vaccines) of providing each PEP dose and the total cost per patient for the two sites are shown in Table 3. Across both sites, the costs per patient fell as the project progressed and higher numbers of patients presented at ABTCs. Dingras, the rural municipality, had a lower average cost for providing PEP per patient than Laoag City (average of $49.02 per patient given PEP compared to $69.72 per patient), which is partly explained by the average number of PEP doses per patient being higher in the city, but also the price per dose of PEP being lower in Dingras (Table 3).

**Figure 2:**
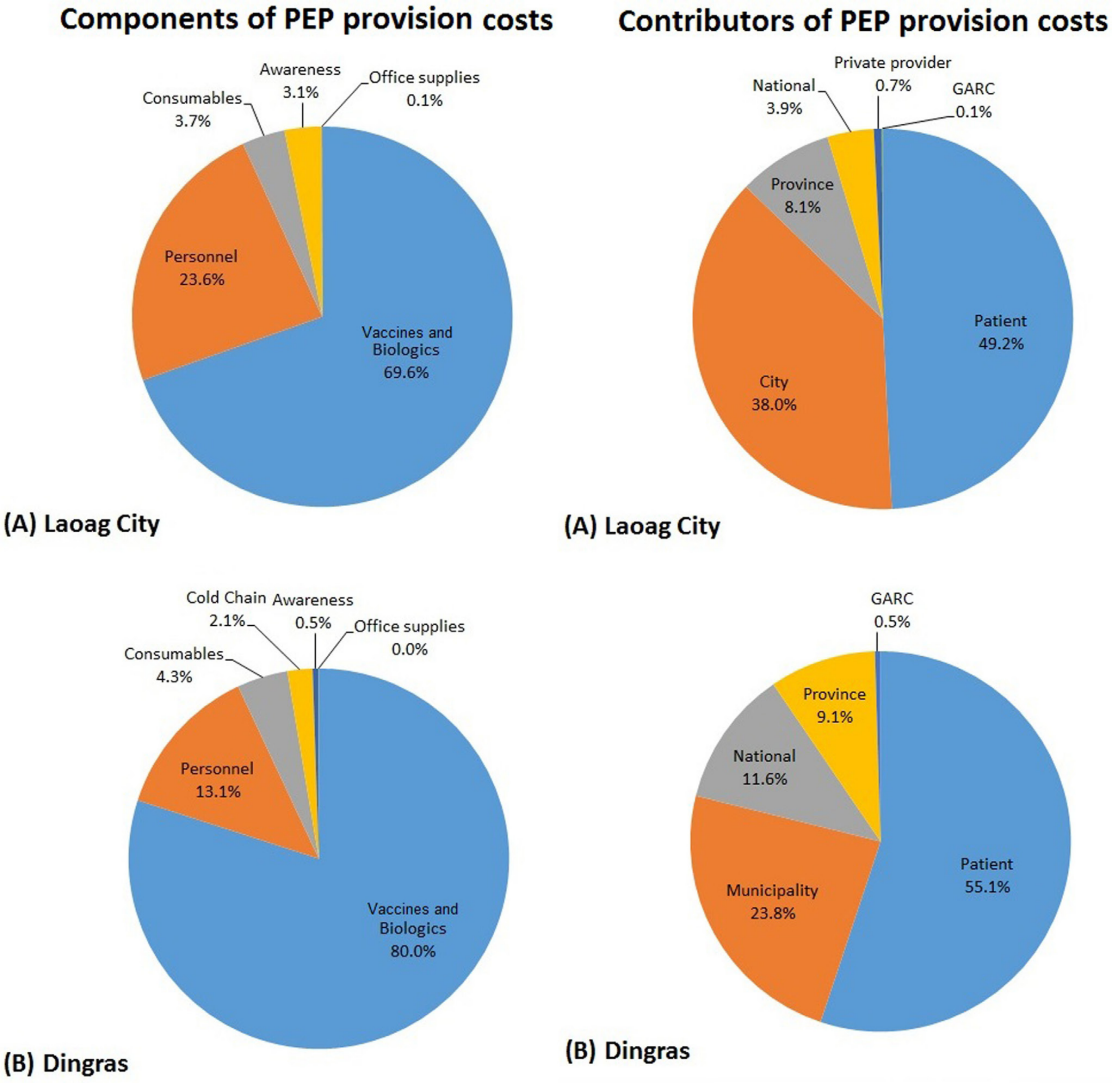
**The cost of providing postexposure prophylaxis (PEP) divided into the different components and between the different project contributors for (A) Laoag City and (B) Dingras Municipality from 2012 to 2014**.

For PEP, the bulk of the costs for both project sites was attributable to the vaccine and biologics (69.6 and 80.0% for Laoag City and Dingras, respectively; Figure 2; Table S3 in Supplementary Material), with personnel the second highest component of cost (23.6 and 13.1%, respectively; Figure 2). The smaller proportion of personnel costs in Dingras compared to that in Laoag City may help to explain why the overall cost per PEP dose was lower.

At the time of this project, PEP was partially subsidized by the government, but patients still provided the most significant amount of the total cost of providing PEP (49.2 and 55.1%, respectively; Figure 2). Project donor contributions were insignificant (a small contribution toward awareness activities), and the bulk of the remaining costs were provided by local government with city and municipality supplying 38.0 and 23.8% of the costs to the sites, respectively (Figure 2).

## Discussion

Governments in Africa and Asia are starting to translate research-led proofs of principle into practical rabies elimination interventions that save lives at scale. Although often not a priority, it is important to document such efforts for the benefit of rabies elimination programs evolving elsewhere.

Before the implementation of the CARE project, sporadic vaccination and emergency interventions produced some reduction in canine rabies case numbers, but failed to reduce them to zero. The CARE project demonstrated that more intensive dog vaccination efforts can break long-term cycles of rabies transmission. Consequently, human and canine case numbers were reduced to zero within 2 years. The trend in human rabies case numbers closely followed that of cases in dogs, as for previous rabies elimination programs (9, 16, 17), re-enforcing the value of focusing effort on dog vaccination. Other parts of the Philippines and other canine rabies-endemic countries could rapidly reduce their human rabies deaths by investing in more comprehensive canine vaccination.

The CARE program benefited from strong legislative and leadership support from the Philippines Government, strong intersectoral collaboration, and the regional and national surveillance systems able to provide a clear assessment of the progress of the project. Such supporting mechanisms facilitate the success and full evaluation of rabies control programs and should be prioritized during planning.

Of the total cost per dog vaccinated, a much higher proportion was attributed to personnel costs than to vaccine. Governments interested in conducting mass dog vaccination must invest in personnel costs and training, not just in the procurement of vaccines if campaigns are to succeed. Other provinces in the Philippines have access to vaccines from the national government, but do not have enough field staff to conduct effective vaccination campaigns, which needs to be addressed. The CARE project’s use of volunteers such as barangay health workers who served as vaccinators, recorders, and community mobilizers could be replicated to enable limited personnel to be more effective and to facilitate higher community engagement, which delivers economies of scale.

One major drawback was the lack of accurate estimates of the total dog population, which seriously limited the estimations of the vaccination coverage, the surveillance effort, and the incidence of rabies in dogs. A number of other rabies control programs have suffered from initial dog population estimates that were too high [e.g., in Tanzania (18)], too low [e.g., KZN and Philippines (19)], or simply uncertain [e.g., Haiti (20)]. Reliable confidence intervals on dog population estimates are difficult to determine, but the conflicting data over coverage suggest that either the most detailed estimation of the dog population was too high (possible if the detailed survey was carried out in unrepresentative sites) or that the reporting vaccinations was not complete.

A valuable improvement for areas with poor data on dog populations would be to utilize mark–recapture assessments to provide estimates of vaccination coverage immediately after the first vaccinations are completed in a location (21, 22). This is labor intensive, but these data can very quickly provide “real-time” vaccination coverage data to guide catch-up or subsequent vaccination campaigns. Combined with accurate dog vaccination numbers, such surveys can also provide immediate feedback on the estimated dog population size.

Despite uncertainty over the exact vaccination coverage achieved, it is clear that the Ilocos Norte CARE project did break the cycle of rabies transmission with likely much lower than recommended (70%) vaccination rates in dogs. Lower than recommended vaccination coverages have also been noted in successful rabies control efforts in KwaZulu-Natal and the Philippines (6).

The recommended coverage of 70% is a conservative value (23), and it is possible that rabies in this province has a relatively low rate of spread. This could allow a modest increase in vaccination coverage achieved to still have a significant impact on transmission, which was suggested as possible in recent modeling studies (24). The estimated 36% of dogs that were kept confined by owners may have contributed to lower rabies transmission rates within the free-roaming dog population. However, the vaccination campaign was expected to have reached mostly owned dogs, and the estimated 22% of dogs without owners may not have been vaccinated. With only two private veterinary practices in Ilocos Norte, large numbers of privately vaccinated dogs are unlikely.

The reported dog cases were only those that were laboratory confirmed, so there is a risk that other cases could be missed. However, surveillance effort was consistent throughout the project, and the case numbers clearly decreased to zero, with monthly negative monitoring reports suggesting that this is still true. Through coordination between animal health and human health sector, animal bites from highly suspect rabid animals are investigated, and all suspect canine rabies cases in recent years have, after observation, been found to be negative.

Even as canine rabies cases fell, bite treatment numbers rose, a trend that has been found elsewhere (8, 9, 25, 26). The impact of educational and other community-based bite prevention activities usually increases the reporting rate of bites (seen here from 2012 to 2014) and could mask any effect of reduced numbers of bites occurring. However, given the extensive awareness exercises conducted in the CARE project, the availability of eight bite treatment centers, and the Philippines’ government subsidy for PEP, a high percentage of patients were expected to seek treatment for bites by the end of the project. It is possible that the decrease in bite consultations between 2014 and 2015 may be a result of reduced number of bites, but this requires further data to confirm.

Lower costs per dog vaccinated with higher throughput and higher human density have been observed in other programs (18, 25), and overall costs fell in the middle and toward the higher end of the range of previously documented costs (27). In this project, a very small proportion of the costs of dog vaccination were from external donors, which is expected to be a good model for ensuring rabies control is sustained beyond the end of the project.

The cost per patient provided with PEP was consistent with other similar full programmatic cost assessments for Africa (18, 25, 26) and elsewhere in the Philippines (25). Interestingly in Ilocos Norte, the rural health center had lower costs on average than in Laoag City, explained by fewer PEP doses received per patient on average, and a lower programmatic cost per dose in Dingras. In Ilocos Norte, a large proportion of the costs were borne by the patients, similar to elsewhere in the Philippines, where patients paid around 50% of the total costs (25). This situation will change as the Philippines national government has agreed to pay for full courses of PEP for patients presenting from 2016 onward (28).

It is impossible to assess the relative value (and therefore cost-effectiveness) of each component of a mixed intervention. We know that canine vaccination is critical to halt rabies transmission, but education and awareness are critical to build trust and participation in the dog vaccination campaign, and to make sure that people seek PEP when needed. The relatively small investment in education campaigns (such as the training of Rabies Speakers in every municipality of Ilocos Norte) was likely a very cost-effective way to increase the program’s impact.

From 2016 onward, annual mass dog vaccination campaigns will be planned and implemented by the provincial government. The established educational initiatives will continue to maintain high awareness and participation in vaccination programs, and the activities at borders, ports, and airports are expected to play an important role in preventing rabies being reintroduced into the province. There is evidence of increased investment in rabies control from the provincial government, which has invested in the renovation of Rabies Diagnostic Laboratories to enhance laboratory biosafety and biosecurity. One lesson learned already being acted upon is capacity building for vaccinators on the use of nets to catch free-roaming dogs to increase vaccination coverage in this high-risk population of dogs. The expertise gained and lesson learned from the CARE project are already being used to revise the National Rabies Program Manual of Operations, the Medium Term Plan which serve as the basis for the national elimination strategy.

Currently, the 38 nationally declared rabies-free areas in the Philippines are all islands. The absence of rabies in animals and humans for several years in Ilocos Norte has proven that rabies elimination at a provincial level in a landlocked area in one of the major islands of the Philippines is feasible. More active surveillance is now needed with an increase in the submission of dog samples to confirm continued rabies freedom. Laboratory confirmation of human cases is not yet carried out, but should be encouraged to enable viral genetic analysis that can differentiate reintroductions from local transmission. Such measures should be included in the national governmental guidelines to enable all areas in the Philippines to benefit from their application.

It is now a challenge to the national government to plan a more progressive zoning approach toward the elimination of rabies across other rabies-endemic provinces in the major islands of Luzon, Visayas, and Mindanao and to prevent incursions into rabies-free provinces.

## Conclusion

A highly intersectoral model and widespread community engagement backed by national government support and surveillance systems provided the necessary boost to rabies control efforts to eliminate canine rabies from the province in a short time period. This occurred despite apparently low dog vaccination coverages. The programmatic costs were comparable to other recently published programmes and similarly showed decreasing costs as efforts were scaled. Donor funding comprised a small amount of the total investment, and local government support is sufficient that the results should be maintained going forward. However, as the province is still surrounded by endemic areas, vaccination and surveillance must be maintained to rapidly respond to possible reintroductions.

## Ethics Statement

Veterinary and human health activities were conducted by the Provincial Veterinary Office and Provincial Health office under the implementation of the government’s rabies elimination program and as such did not undergo a separate ethics review. All services provided were bound by Republic Act 9482 “Anti-Rabies Act of 2007,” Republic Act 6713 “Code of Conduct and Ethical Standards for Public Officials and Employees,” and Republic Act 9268 “The Philippine Veterinary Medicine Act of 2004.” The data used in the study were based on records collated by the provincial veterinary and health offices.

## Author Contributions

LV and SJ oversaw the Ilocos Norte CARE project; AA, MC, DL, and RG-B collected and analyzed the data; and LT and LN lead the manuscript preparation. All authors contributed to drafting and editing of the manuscript and approved the final version.

## Conflict of Interest Statement

The authors declare that the research was conducted in the absence of any commercial or financial relationships that could be construed as a potential conflict of interest.

## References

[1] Lembo T Attlan M Bourhy H Cleaveland S Costa P de Balogh K Renewed global partnerships and redesigned roadmaps for rabies prevention and control. Vet Med Int (2011) 2011:923149. 10.4061/2011/923149 21776359 PMC3135331

[2] Hampson K Coudeville L Lembo T Sambo M Kieffer A Attlan M Estimating the global burden of endemic canine rabies. PLoS Negl Trop Dis (2015) 9(4):e0003709. 10.1371/journal.pntd.0003709 25881058 PMC4400070

[3] Lembo T Hampson K Kaare MT Ernest E Knobel D Kazwala RR The feasibility of canine rabies elimination in Africa: dispelling doubts with data. PLoS Negl Trop Dis (2010) 4(2):e626. 10.1371/journal.pntd.0000626 20186330 PMC2826407

[4] Cleaveland S Beyer H Hampson K Haydon D Lankester F Lembo T The changing landscape of rabies epidemiology and control. Onderstepoort J Vet Res (2014) 81(2):E1–8. 10.4102/ojvr.v81i2.731 25005807 PMC7612516

[5] WHO. Rabies: Rationale for Investing in the Global Elimination of Dog-Mediated Human Rabies. World Health Organization (2015). Available from: http://apps.who.int/iris/bitstream/10665/185195/1/9789241509558_eng.pdf

[6] WHO, OIE. Global elimination of dog-mediated human rabies: the time is now! Report of the Rabies Global Conference, 2015 Dec 10-11, Geneva, Switzerland. Paris: World Health Organization and World Organisation for Animal Health (2016). Available from: http://www.oie.int/fileadmin/Home/eng/Media_Center/docs/pdf/Rabies_portal/EN_RabiesConfReport.pdf

[7] Vigilato MA Clavijo A Knobl T Silva HM Cosivi O Schneider MC Progress towards eliminating canine rabies: policies and perspectives from Latin America and the Caribbean. Philos Trans R Soc Lond B Biol Sci (2013) 368(1623):20120143. 10.1098/rstb.2012.0143 23798691 PMC3720041

[8] Harischandra PAL Gunesekera A Janakan N Gongal G Abela-Ridder B Sri Lanka takes action towards a target of zero rabies death by 2020. WHO South East Asia J Public Health (2016) 5(2):113–6. 10.4103/2224-3151.206247 28607238

[9] Lapiz S Miranda M Garcia R Daguro L Paman M Madrinan F Implementation of an intersectoral program to eliminate human and canine rabies: The Bohol Rabies Prevention and Elimination Project. PLoS Negl Trop Dis (2012) 6(12):e1891. 10.1371/journal.pntd.0001891 23236525 PMC3516573

[10] ASEAN/OIE. The South-East Asia Dog Rabies Elimination Strategy. (2013). Available from: http://www.rr-asia.oie.int/fileadmin/SRR_Activities/SEA_Rabies_Strategy_-_OIE_Final_Draft.pdf

[11] OIE. Global Strategic Framework for the Elimination of Dog-Mediated Human Rabies. (2016). Available from: http://www.oie.int/for-the-media/press-releases/detail/article/global-strategic-framework-for-the-elimination-of-dog-mediated-human-rabies/

[12] Wikipedia. Ilocos Norte. (2016). Available from: https://en.wikipedia.org/wiki/Ilocos_Norte

[13] Philippine Statistics Authority. Population of Region I – Ilocos Region. (2016). Available from: https://www.psa.gov.ph/content/population-region-i-ilocos-region-based-2015-census-population

[14] Philippine Statistics Authority. Poverty Incidence among Filipinos Registered at 26.3%, as of First Semester of 2015 – PSA. (2016). Available from: https://www.psa.gov.ph/content/poverty-incidence-among-filipinos-registered-263-first-semester-2015-psa

[15] Philippine Statistics Authority. 2012 Annual Average Income and Expenditure by Region and Province. (2013). Available from: https://www.psa.gov.ph/content/2012-annual-average-income-and-expenditure-region-and-province

[16] Ortiz-Prado E Ponce-Zea J Ramirez D Stewart-Ibarra AM Armijos L Yockteng J Rabies epidemiology and control in Ecuador. Glob J Health Sci (2015) 8(3):113–21. 10.5539/gjhs.v8n3p113 26493436 PMC4804063

[17] Taylor LH Nel LH Global epidemiology of canine rabies: past, present, and future prospects. Vet Med (2015) 6:361–71. 10.2147/VMRR.S51147 PMC6067664 30101121

[18] Hatch B Anderson A Sambo M Maziku M McHau G Mbunda E Towards canine rabies elimination in South-Eastern Tanzania: assessment of health economic data. Transbound Emerg Dis (2016). 10.1111/tbed.12463 26916104

[19] WHO. Report of the Fourth meeting of the International Coordinating Group of the Bill & Melinda Gates Foundation–World Health Organization Project on Eliminating Human and Dog Rabies Cebu City, Philippines (2013). Available from: http://apps.who.int/iris/bitstream/10665/79216/1/WHO_HTM_NTD_NZD_2013.1_eng.pdf?ua=1

[20] Millien MF Pierre-Louis JB Wallace R Caldas E Rwangabgoba JM Poncelet JL Control of dog mediated human rabies in Haiti: no time to spare. PLoS Negl Trop Dis (2015) 9(6):e0003806. 10.1371/journal.pntd.0003806 26110845 PMC4481514

[21] Lechenne M Oussiguere A Naissengar K Mindekem R Mosimann L Rives G Operational performance and analysis of two rabies vaccination campaigns in N’Djamena, Chad. Vaccine (2016) 34(4):571–7. 10.1016/j.vaccine.2015.11.033 26631415

[22] Gibson AD Ohal P Shervell K Handel IG Bronsvoort BM Mellanby RJ Vaccinate-assess-move method of mass canine rabies vaccination utilising mobile technology data collection in Ranchi, India. BMC Infect Dis (2015) 15:589. 10.1186/s12879-015-1320-2 26715371 PMC4696259

[23] Coleman PG Dye C. Immunization coverage required to prevent outbreaks of dog rabies. Vaccine (1996) 14(3):185–6. 10.1016/0264-410X(95)00197-9 8920697

[24] Fitzpatrick MC Shah HA Pandey A Bilinski AM Kakkar M Clark AD One health approach to cost-effective rabies control in India. Proc Natl Acad Sci U S A (2016) 113(51):14574–81. 10.1073/pnas.1604975113 27994161 PMC5187709

[25] Miranda LM Miranda ME Hatch B Deray R Shwiff S Roces MC Towards canine rabies elimination in Cebu, Philippines: assessment of health economic data. Transbound Emerg Dis (2017) 64(1):121–9. 10.1111/tbed.12350 25885005

[26] Shwiff SA Hatch B Anderson A Nel LH Leroux K Stewart D Towards canine rabies elimination in KwaZulu-Natal, South Africa: assessment of health economic data. Transbound Emerg Dis (2016) 63(4):408–15. 10.1111/tbed.12283 25414096

[27] Elser JL Hatch BG Taylor LH Nel LH Shwiff SA. Towards canine rabies elimination: economic comparisons of three project sites. Transbound Emerg Dis (2017) 1–11. 10.1111/tbed.12637 28299897

[28] Government of the Philippines. Free Anti-Rabies Vaccines in 2016. (2016). Available from: http://www.gov.ph/2016/01/05/free-anti-rabies-vaccines-in-2016/

