# Supplementary material for: The Ilocos Norte Communities against Rabies Exposure Elimination Project in the Philippines: Epidemiological and Economic Aspects
Source: Front Vet Sci. 2017 Apr 24;4:54. doi: 10.3389/fvets.2017.00054 (PMC5402182; doi:10.3389/fvets.2017.00054)
Supplement: Supplementary file 1 [file Data_Sheet_1.DOCX]

**The Ilocos Norte Communities Against Rabies Exposure (CARE) Elimination Project in the Philippines: Epidemiological And Economic Aspects**

Loida M. Valenzuela, Sarah I. Jayme, Anna Charinna B. Amparo, Louise H. Taylor, Maria Pinky Z. Dela Cruz, Dianne A. Licuan, Rosebelle Gamal-Bitao, Louis H. Nel

**Supplementary Data Tables**

**Table S1A**. **Total dogs per 10 persons and total owned dogs per 10 persons as a function of human population density and corresponding dog:human ratios.** Source: J. Boone, unpublished data

| Human Population / km^2^ | Total Dogs / 10 people | Owned Dogs / 10 people | Dog:human ratio | Owned Dog:human ratio |
| --- | --- | --- | --- | --- |
| 0 – 999 | 0.493 | 0.362 | 1: 2.03 | 1: 2.76 |
| 1,000 – 4,999 | 0.392 | 0.328 | 1: 2.55 | 1: 3.05 |
| > 5,000 | 0.445 | 0.380 | 1: 2.25 | 1: 2.63 |
| **OVERALL** | **0.447** | **0.348** | **1: 2.24** | **1:2.87** |

**Table S1B**. **Estimates of dog population sub-categories as a function of human population density.** Dog: human ratios in each category are shown in parentheses beside each associated dog population estimate. Totals shown may be affected slightly by rounding errors. Source: J. Boone, unpublished data.

| Human Population / km^2^ | Unowned Dogs | Owned, Free-Roaming Dogs | Owned, Confined Dogs | TOTAL Dogs |
| --- | --- | --- | --- | --- |
| 0 – 999 | 41,611 (1:7.63) | 69,064  (1:4.61) | 45,840  (1:6.94) | **156,514** |
| 1,000 – 4,999 | 17,273 (1:15.62) | 42,861  (1:6.29) | 45,797  (1:5.88) | **105,931** |
| > 5,000 | 2,338 (1:15.62) | 3,268  (1:11.23) | 10,640  (1:3.43) | **16,246** |
| **TOTAL** | **61,222** | **115,193** | **102,276** | **278,691** |

**Table S2A Dog vaccination costs for Laoag City costs divided by category of expenditure and course of funding.** All figures are in Philippines Pesos

| **CATEGORY** | **PAID FOR BY** | **2012** | **2013** | **2014** | **TOTAL** |
| --- | --- | --- | --- | --- | --- |
| Personnel | GARC | - | 44,324 | 48,224 | 92,549 |
| Personnel | Province | 213,904 | 221,797 | 221,797 | 657,499 |
| Personnel | City | 534,612 | 534,618 | 528,722 | 1,597,952 |
| Vaccine | GARC | 3,940 | - | 119,228 | 123,167 |
| Vaccine | Region | 7,295 | - | - | 7,295 |
| Vaccine | Province | 100,000 | - | - | 100,000 |
| Vaccine | City | 35,000 | 35,000 | 35,000 | 105,000 |
| Vaccine | Other donor (OIE) | - | 97,965 | - | 97,965 |
| Vaccine consumables | GARC | - | 52,370 | 27,020 | 79,390 |
| Vaccine consumables | Province | 827 | 383 | 974 | 2,184 |
| Vaccine consumables | City | 38,328 | 38,328 | 38,328 | 114,984 |
| Transport | GARC | 5,000 | 5,000 | 5,000 | 15,000 |
| Transport | Province | - | - | 6,580 | 6,580 |
| Transport | City | - | - | - | - |
| Cold chain | GARC | 3,841 | - | - | 3,841 |
| Cold chain | Province | 600 | - | 210 | 810 |
| Cold chain | City | 1,300 | - | - | 1,300 |
| PrEP for vaccinators | GARC | 5,976 | - | - | 5,976 |
| Awareness and social mobilization | GARC | 154,493 | 9,840 | 12,985 | 177,318 |
| Awareness and social mobilization | National | - | - | 6,628 | 6,628 |
| Awareness and social mobilization | Province | - | - | 1,800 | 1,800 |
| Office supplies | Province | 1,889 | - | - | 1,889 |
| Diagnostics | Province | 3,361 | 859 | 1,706 | 5,926 |
| **TOTAL** |  | 1,110,365 | 1,040,485 | 1,054,202 | 3,205,053 |

**Table S2B Dog vaccination costs for Dingras Municipality costs divided by category of expenditure** All figures are in Philippines Pesos

| **CATEGORY** | **PAID FOR BY** | **2012** | **2013** | **2014** | **TOTAL** |
| --- | --- | --- | --- | --- | --- |
| Personnel | GARC | - | 15,642 | 15,642 | 31,284 |
| Personnel | Province | 75,488 | 78,273 | 78,273 | 232,034 |
| Personnel | Municipality | 469,745 | 469,745 | 469,745 | 1,409,234 |
| Vaccine | GARC | - | - | 21,000 | 21,000 |
| Vaccine | Region | 7,473 | - | - | 7,473 |
| Vaccine | Province | - | - | - | - |
| Vaccine | Municipality | 7,475 | 30,000 | - | 37,475 |
| Vaccine | Others (OIE) | - | 53,025 | - | 53,025 |
| Vaccine consumables | GARC | - | 16,000 | - | 16,000 |
| Vaccine consumables | Province | - | - | 81 | 81 |
| Vaccine consumables | Municipality | 15,810 | 36,480 | 36,480 | 88,770 |
| Transport | Municipality | 600 | 15,000 | 15,000 | 30,600 |
| Cold chain | GARC | 600 | - | - | 600 |
| Cold chain | Province | 600 | - | - | 600 |
| Cold chain | Municipality | 750 | - | - | 750 |
| PrEP for vaccinators | GARC | 234 | - | - | 234 |
| Awareness and social mobilization | GARC | 12,182 | 36,480 | 10,800 | 59,462 |
| Awareness and social mobilization | Province | - | - | 1,800 | 1,800 |
| Diagnostics | Province | 200 | 400 | - | 600 |
| TOTAL |  | 591,155 | 751,045 | 648,821 | 1,991,021 |

**Table S3A Laoag City PEP costs by category of expenditure and source of funding.** All figures are in Philippines Pesos

| **CATEGORY** | **PAID FOR BY** | **2012** | **2013** | **2014** | **TOTAL** |
| --- | --- | --- | --- | --- | --- |
| Personnel | Province | 153,245 | 184,022 | 208,401 | 545,668 |
| Personnel | City | 368,232 | 368,232 | 368,232 | 1,104,696 |
| Personnel | Private provider | 24,378 | 24,378 |  | 48,757 |
| Awareness activities | GARC |  |  | 6,365 | 6,365 |
| Awareness activities | City |  |  | 219,700 | 219,700 |
| Biologics | National | 37,055 | 59,700 | 187,310 | 284,065 |
| Biologics | Province | 6,400 | 20,200 | 4,000 | 30,600 |
| Biologics | City | 554,800 | 339,900 | 500,250 | 1,394,950 |
| Biologics | Patient | 935,500 | 1,221,092 | 1,150,820 | 3,307,412 |
| Consumables | Province | 702 | 702 | 702 | 2,105 |
| Consumables | City | 6,090 | 6,090 | 6,090 | 18,270 |
| Consumables | Patient | 69,270 | 72,120 | 101,910 | 243,300 |
| Office supplies | Province | 1,186 | 1,186 | 1,436 | 3,807 |
| **TOTAL** |  | 2,156,857 | 2,297,622 | 2,755,215 | 7,209,695 |

**Table S3B Dingras Municipality PEP costs by category of expenditure and source of funding.** All figures are in Philippines Pesos

| **CATEGORY** | **PAID FOR BY** | **2,012** | **2,013** | **2,014** | **TOTAL** |
| --- | --- | --- | --- | --- | --- |
| Personnel | Province | 33,077 | 43,939 | 43,939 | 120,955 |
| Personnel | Municipality | 18,170 | 18,170 | 18,170 | 54,509 |
| Awareness activities | GARC |  |  | 6,365 | 6,365 |
| Biologics | National | 27,420 | 20,075 | 79,935 | 127,430 |
| Biologics | Municipality | 0 | 114,850 | 147,900 | 262,750 |
| Biologics | Patient | 99,113 | 163,120 | 421,925 | 684,158 |
| Consumables | Municipality | 106 | 1,624 | 106 | 1,836 |
| Consumables | Patient | 6,090 | 14,070 | 36,120 | 56,280 |
| Cold chain | National |  | 28,542 |  | 28,542 |
| Office supplies | Province | 0 | 0 | 668 | 668 |
| **TOTAL** |  | 183,976 | 404,390 | 755,128 | 1,343,494 |
